# Associations Between Youth Sport Participation and Bone, Muscle, and Fat in Adulthood: Iowa Bone Development Study

**DOI:** 10.3390/ijerph22030416

**Published:** 2025-03-12

**Authors:** Soyang Kwon, Fátima Baptista, Steven M. Levy, Indranil Guha, Punam K. Saha, Kathleen F. Janz

**Affiliations:** 1 Department of Emergency Medicine, Buehler Center for Health Policy and Economics, Northwestern University, Chicago, IL 60208, USA; 2 CIPER, Faculdade de Motricidade Humana, Universidade de Lisboa, 1499-002 Estrada da Costa, Portugal; fbaptista@fmh.ulisboa.pt; 3 Department of Preventive and Community Dentistry, University of Iowa, Iowa City, IA 52242, USA; steven-levy@uiowa.edu; 4 Department of Electrical and Computer Engineering, University of Iowa, Iowa City, IA 52242, USA; indranil-guha@uiowa.edu (I.G.); punam-saha@uiowa.edu (P.K.S.); 5 Department of Health and Human Physiology, University of Iowa, Iowa City, IA 52242, USA; kjanz@uiowa.edu

**Keywords:** body composition, bone strength, physical activity, cohort study, dual-energy X-ray absorptiometry, finite element analysis, high-resolution multi-row detector computed tomography

## Abstract

The objective of this study was to characterize bone, muscle, and fat measurements in early adulthood by youth sport participation. The study sample included 328 Iowa Bone Development Study participants (184 females). Organized sport participation was longitudinally assessed (14 times on average) using a physical activity questionnaire from age 6 to 17 years. At age 23 years, bone, lean mass (a proxy measure of muscle mass), and fat mass were assessed using dual-energy X-ray absorptiometry (DXA). Tibial bone stiffness, a bone strength indicator, was determined using high-resolution multi-row detector computed tomography (CT) and Finite Element Analysis. Longitudinal youth sport participation patterns were categorized into consistent participation, drop-out, and no participation. Sex-specific multivariable linear regression analyses were conducted to examine the associations between youth sport participation patterns and bone mineral content (BMC), lean mass index (LMI), fat mass index (FMI), BMC-to-lean ratio, lean-to-fat ratio, and tibial bone stiffness at age 23 years. After adjusting for covariates, males in the consistent youth sport participation and drop-out groups had 377 g and 192 g higher BMC, 1.6 kg/m^2^ and 1.5 kg/m^2^ higher LMI, and 112 kN/mm and 76 kN/mm higher bone stiffness at age 23, compared to males in the no-participation group (*p* < 0.01). Females in the consistent youth sport participation group had 1.4 kg/m^2^ lower FMI at age 23, compared to females in the no-participation group (*p* = 0.04). The BMC-to-lean ratio was higher among males (*p* = 0.02) and females (*p* < 0.01) in the consistent participation group, compared to their counterparts in the no-participation group; the lean-to-fat ratio also tended to be higher in males (*p* = 0.06) and females (*p* = 0.11). This study suggests sex differences in the benefits of youth sport participation on adulthood body compartments: healthier bone and muscle for males and healthier body fat for females. This study provides evidence to support the promotion of youth sport participation for healthy body composition later in life.

## 1. Introduction

Early adulthood is a critical period for achieving the healthy development of bone, muscle, and fat tissue (conceptualized as a three-compartment model of body composition) [1]. Peak levels of bone and muscle mass are reached in early childhood [2,3] and low peak bone and muscle mass in that period increase the risks of osteoporosis, physical dysfunction, sarcopenia, and cardiovascular disease later in life [4,5,6,7,8,9,10,11,12,13]. Beyond bone mass that characterizes the amount of bone, bone strength refers to the resistance of a specific bone to deformation and is determined by a combination of both material and structural properties [14,15]. Bone strength has been recognized as an important indicator of bone fracture [16,17].

Since young adults are generally considered one of the healthiest groups in the life course, they are often overlooked in physical health studies and seldom prioritized for disease prevention strategies [18]. However, health risks increase in early adulthood among many individuals. For example, the obesity incidence is higher in the early adulthood period than in adolescence or any other adulthood period [19,20,21,22,23]. Prior investigations, including those from the Iowa Bone Development Study (IBDS) have demonstrated the significant contributions of youth participation in sports and physical activity to the acquisition of bone and muscle mass and the prevention of excessive fat accumulation from childhood to early adulthood [24,25,26,27,28], with potential sex differences in the contributions [29,30]. However, these studies did not simultaneously examine the three main body compartments (bone, muscle, and fat) within an integrated framework [1]. The evidence can be strengthened by improving measurement methodologies with high-resolution body compartment measures and advanced analytical approaches, such as finite element analysis (FEA) for bone strength assessment. A critical knowledge gap remains in understanding how youth sports and physical activity impact the development of each compartment and their inter-relationships in adulthood.

Expanding on our prior IBDS investigations and uniquely applying a three-compartment model of body composition [1], the primary aim of the present study was to characterize bone, muscle, and fat mass, as well as ratios of these compartments and bone strength in early adulthood by youth sport participation trajectories. We hypothesized that individuals who participated in youth sports throughout childhood and adolescence would have a healthier body composition and bone strength in early adulthood, compared to those who did not. A secondary study aim was to identify specific types of youth sport associated with bone and muscle measurements. Based on the contemporary literature on bone adaptation suggesting that bone responds best to movements that are dynamic, short, applied quickly, variable in load, and of high load magnitude [31], we hypothesized that participation in high-power youth sports that involve quick but high-loading movements, such as volleyball and basketball, is associated with stronger bone and muscle.

## 2. Materials and Methods

### 2.1. Study Sample

The study sample was IBDS participants recruited from a subset of the Iowa Fluoride Study birth cohort. The Iowa Fluoride Study cohort included 1882 full-term newborns recruited from 8 hospital postpartum wards in Iowa, USA, between 1992 and 1995 [32]. Iowa Fluoride Study participants were invited to participate in IBDS when the child’s age was approximately 5 years. IBDS baseline examinations were conducted at age 5 years, and the last follow-up visits occurred at age 23 years. A more detailed sample description can be found in our prior publications [33,34]. For this report, the eligibility criteria were Iowa Bone Development Study participants and completion of a body composition assessment at age 23 years (*n* = 328; 184 females). The University of Iowa Institutional Review Board approved the study protocol (IRB ID#199112665). Informed written consent was obtained from each participant. This study adheres to the Declaration of Helsinki.

### 2.2. Exposures

The primary exposure was youth sport participation. We examined youth sport participation by estimating distinct sport participation trajectory patterns from childhood through adolescence (6 to 17 years of age). Because the definition of sports by the National Youth Sports Strategy (NYSS) [35] includes not only competitive sports but also recreational sports that are played as a team or an individual, we included organized sports (defined as coached sports or lessons) through either competitive (e.g., interscholastic sports) or recreational participation (e.g., swimming lessons) [36].

Youth sport participation was assessed using a physical activity questionnaire (PAQ) [36] sent via mail or administered by trained research staff during a research clinic visit every six months. Over time, two versions of the PAQ were used. The first version (V1) was a parent-reported PAQ for participants younger than 11 years of age. The V1 PAQ asked “Did your child participate in any of the following organized sports during the past six months?” Responses included baseball, basketball, soccer, gymnastics, dance, swimming, and other. Responses of “other” were reviewed to capture other sports that were not listed [37]. Based on the V1 PAQ responses, children who participated in at least one organized sport in the past six months were categorized as participating in sports. The second version (V2), adapted from the PAQs for older children (PAQ-C) and adolescents (PAQ-A) [38,39], was a self-reported PAQ for participants aged 11 years or older.

The V2 PAQ listed 16 sports and other physical activities (e.g., walking briskly for exercise) and asked about the frequency of participation in the previous seven days and whether the activity was organized (yes/no). If a respondent reported participating in at least one organized sport at least once a week, the respondent was categorized as participating in sports. More detailed information on the definition of youth sport participation is available in our prior publications [36,37]. Using repeatedly assessed sport participation data (measured 14 times on average between 6 and 17 years of age), our prior group-based trajectory analysis [40] identified three distinct sport participation trajectory patterns (consistent participation, drop-out, and no participation) and assigned each participant into one of the patterns based on the highest likelihood [36,37].

In addition, we categorized high school (grades 9 to 12) interscholastic sport participation based on the mechanical loading of the axial (weight-bearing) skeleton, as studies [41,42] have suggested sport-specific associations with bone and muscle mass by mechanical loading levels, specifically that power movements are more osteogenic than lower, slower intensity movements. Interscholastic high school sport participation was assessed using a self-reported questionnaire at age 17 years. Those who reported participating in interscholastic sports in high school were asked to report the number of years (seasons) they participated in each of 16 different interscholastic sports.

To categorize sport types by mechanical loading, we used the power classification developed based on the ground reaction forces and peak strain scores of each sport as reported in prior work [26,43]. Participants were then categorized into one of three groups: (1) high-power sports if they participated in at least two years of a high-power sport (e.g., basketball, cheerleading/poms, football, soccer, volleyball); (2) other sports if they participated in non-high-power sports (e.g., baseball, softball, tennis, wrestling) and/or participated in only one year of a high-power sport; (3) no sports, if they did not participate in interscholastic sports. The ≥2-year criteria for the high-power sports group was to ensure adequate exposure to the sport that impacts bone development [43].

### 2.3. Outcomes

The outcome variables of interest included bone mineral content (BMC; g), lean mass index (LMI; kg/m^2^), fat mass index (FMI; kg/m^2^), BMC-to-lean ratio (g/kg), and lean-to-fat ratio (kg/kg) at age 23 years. Lean body mass was used as a proxy indicator of muscle mass. We also included bone stiffness as an outcome variable to examine bone strength.

At age 23, participants underwent a whole-body dual X-ray absorptiometry (DXA; Hologic Discovery A model; Hologic Inc., Bedford, MA, USA) to determine body composition [44]. All scans were conducted by one of three technicians certified by the International Society of Clinical Densitometry. Pregnant females were excluded from the DXA examination. Software version 12.6 and the fan-beam mode were used for all scan acquisitions. Quality control scans were performed daily using the Hologic spine phantom. Our laboratory’s precision error for BMC measurements is low (coefficient of variation <1% for quality control scans performed daily using the Hologic phantom). The detailed DXA procedure is described in our prior publication [43]. BMC, lean mass, and fat mass were derived from the scan using the Hologic APEX 4.0 software. We used whole-body-less head BMC (g) as a BMC indicator. LMI was calculated as whole-body lean mass divided by height squared (kg/m^2^). FMI was calculated as whole-body fat mass divided by height squared (kg/m^2^) [45]. BMC-to-lean ratio (BMC divided by lean mass; g/kg) and lean-to-fat mass ratio (lean body mass divided by fat mass; kg/kg) were also calculated [45].

Participants also underwent computed tomography (CT) imaging of the distal tibia using a Siemens FORCE CT scanner (Siemens Healthineers, Forchheim, Germany; 120 kV, 100 effective mAs). Images were generated at 200 μm slice spacing and approximately 170 μm pixel resolution. Trained technicians conducted CT scans. A calibration phantom scan was used to convert CT intensity values into bone mineral density (mg/cc). The nonlinear continuum FEA-based stiffness measure has been reported to be associated with bone micro-structural quality at in vivo imaging resolution [46,47]. A validated nonlinear continuum FEA method was applied to compute bone stiffness (kN/mm) at the 4–6% distal tibia location [48].

### 2.4. Other Variables

To account for the effects of body size and bone measures at baseline, we considered body mass index (BMI) percentile and DXA-based BMC at age five years as confounders. Trained nurses measured participants height and mass in the University of Iowa General Clinical Research Center during a study visit. Based on our prior IBDS analyses for bone and body composition outcomes [25,26,27], we considered education level, energy intake, and ActiGraph GT3X accelerometer-measured moderate- and vigorous-intensity physical activity (MVPA) [49] at age 23 years as confounders to account for the effects of concurrent sociodemographic status, energy intake, and physical activity. Total energy intake was estimated based on food frequency questionnaire data and categorized into sex-specific tertile groups. For 29 participants who did not complete an accelerometer measurement, we used the PAQ data (weekly MVPA minutes) at age 23 years to impute accelerometer-based MVPA based on a sex- and age-adjusted linear regression model.

### 2.5. Statistical Analysis

All analyses were conducted using SAS 9.4 (Cary, NC, USA). Because body composition is known to differ by biological sex [10], sex-specific analyses were conducted. Because only 55% of the IBDS cohort were included in this analysis, we conducted chi-squared tests and *t*-tests to compare demographic distributions between those who were included and those who were not.

Descriptive analyses of the study variables were conducted. The normal distribution of the study variables was tested using Shapiro–Wilk tests. Results were presented as mean ± standard deviation [SD]. An analysis of variance (ANOVA) and post hoc Tukey’s tests were performed to compare study variables between the youth sport participation trajectory patterns and between the high school sport participation groups. We selected the outcome variables that were found to be statistically significantly associated with youth sport participation patterns in bivariate analyses for subsequent multivariable linear regression analyses.

Five multivariable linear regression models were built to predict the selected outcome variables by youth sport participation patterns: consistent participation, drop-out, vs. no participation [36,37]. For the FMI variable that was not normally distributed, log-transformed FMI was used for regression analysis. Other outcome variables were normally distributed. All models were adjusted for age in years, education, and MVPA (an additional 10 min/day). The BMC outcome was additionally adjusted for baseline BMC and concurrent height. The bone stiffness outcome was additionally adjusted for baseline BMC. The LMI, BMC-to-lean ratio, and lean-to-fat ratio outcomes were additionally adjusted for the baseline BMI percentile. The FMI outcome was additionally adjusted for baseline BMI percentile and concurrent energy intake. A significance level was corrected as 0.01 (0.05 ÷ 5) using the Bonferroni method for multiple comparisons. Collinearity was tested using variance influence factors and a correlation matrix.

The female sample (73 in the consistent participation group, 66 in the drop-out group, and 45 in the no-participation group) provided 74% power to detect a 0.5 effect size between the consistent participation and no-participation groups and 73% power to detect a 0.5 effect size between the drop-out and non-participation groups. The male sample (56 in the consistent participation group, 53 in the drop-out group, and 35 in the no-participation group) provided 63% power to detect a 0.5 effect size between the consistent participation and no-participation groups and 62% power to detect a 0.5 effect size between the drop-out and no-participation groups.

## 3. Results

Among 630 IBDS participants, the included (*n* = 328) and excluded (*n* = 302) participants had similar demographic distributions: self-identified non-Hispanic White race = 96% vs. 94% (*p* = 0.25), female = 56% vs. 51% (*p* = 0.20), and maternal 4-year college education = 74% vs. 72% (*p* = 0.53). The average baseline BMI was 21.0 kg/m^2^, for the included and the excluded (*p* = 0.97). Of the 328 included participants, 58.5% reported having completed a 4-year college degree education, and 61.6% reported having a full-time job at the age 23-year assessment. For living arrangements, 42.8% lived with roommate(s), 18.0% lived with parent(s), and 14.8% lived alone.

As presented in Table 1, the average baseline BMC and BMI percentiles, as well as birth weight, did not significantly differ among the three youth sport participation groups. Among males at age 23 years, the consistent youth sport participation group had a significantly higher BMC, LMI, BMC-to-lean ratio, lean-to-fat ratio, and bone stiffness compared to the no-participation group.

Among females at age 23 years, the consistent youth sport participation group had a significantly higher BMC, BMC-to-lean ratio, and lean-to-fat ratio, and significantly lower FMI, compared to the no-participation group. An examination by interscholastic high school sport types (Table 2) showed that males and females in the high-power sport group had a significantly higher BMC at age 23 compared to the other sport or no sport group. Males in the high-power sport group had a higher BMC-to-lean ratio, lean-to-fat ratio, and bone stiffness at age 23 years, compared to males in the no sport group.

After adjusting for the covariates (Table 3), males in the consistent youth sport participation and drop-out groups had 377 g and 192 g higher BMC, 1.6 kg/m^2^ and 1.5 kg/m^2^ higher LMI, and 112 kN/mm and 76 kN/mm higher bone stiffness at age 23, compared to males in the no-participation group (*p* < 0.01). Females in the consistent youth sport participation group had 1.4 kg/m^2^ lower FMI at age 23, compared to females in the no-participation group (*p* = 0.04).

After adjusting for the covariates (Table 4), the BMC-to-lean ratio was higher among males (*p* = 0.02) and females (*p* < 0.01) in the consistent participation group, compared to their counterparts in the no-participation group. The lean-to-fat ratio also tended to be higher, but it did not reach a significance level in both males (*p* = 0.06) and females (*p* = 0.11). After adjusting for the covariates, males in the high-power interscholastic sport group showed a higher BMC, BMC-to-lean ratio, lean-to-fat ratio, and bone stiffness at age 23, compared to males in the no interscholastic sport group (Supplementary Table S1). Females in the high-power interscholastic sport group tended to have a higher BMC (*p* = 0.06).

## 4. Discussion

This study of primarily non-Hispanic White American participants found that males and females who consistently participated in youth sports had more favorable relationship measures between body compartments, such as the BMC-to-lean ratio and lean-to-fat ratio, in early adulthood, compared to their counterparts who did not participate in youth sports. However, how these favorable ratios were achieved differed by sex. Males who participated in youth sports either consistently throughout childhood and adolescence or dropped out presented a significantly higher BMC and lean mass in early adulthood, compared to males who did not participate in youth sports. In contrast, the body fat of males did not differ by youth sport participation patterns. Females who consistently participated in youth sports had significantly lower body fat in early adulthood compared to females who did not participate in youth sports, while the lean mass of females did not differ by youth sport participation patterns. This study also revealed that youth sport participation, particularly high-power interscholastic sport participation, was beneficial for the acquisition of bone mass for both males and females. In contrast, its benefits on bone strength were significant only for males but not for females.

This is one of the first studies to examine the independent and combined relationship between non-invasive, high-quality indicators of the three body compartments (bone, muscle, fat) and the effects of the volume and intensity of mechanical load of youth sport participation. Each of the bone, muscle, and fat compartments and their inter-relationships are critical to maintaining healthy physiological systems. Our three-body compartment approach extends the understanding of the effects of youth sport and physical activity participation on body composition development beyond concerns for obesity. The findings from this study support a sex difference in the effects of volume and intensity of youth sport participation on the development of these body compartments, which has been suggested in prior studies [29,30]. Males who consistently participate in youth sports and even males who initially participate and then drop out would have healthier muscle mass, bone mass, and bone strength later in adulthood, but less so for body fat. On the other hand, females who consistently participate in youth sports would have healthier body fat levels in early adulthood, and females who participated in high-power interscholastic sports could achieve healthier levels of bone mass in early adulthood; but less so for bone strength or muscle mass. Despite the sex differences, youth sport participation ultimately contributes to achieving a healthy bone-to-lean ratio and lean-to-fat ratio in adulthood for both males and females [50].

This study demonstrated the benefits of youth sport participation on bone mass among males. However, its benefits were found to be smaller among females, which is consistent with a review [51] that concluded that a positive association between physical activity during adolescence and BMC in early adulthood was more consistently reported among males than females. When we further examined bone strength, the benefit of youth sport participation on bone strength was only observed among males, but not among females. Consistent participation in sports during childhood and adolescence may be important to differentiate their body composition in early adulthood among females, while participation in high power sports may be a determinant to differentiate both their body composition and bone strength among males. Given females’ lower capacity to develop muscle and bone, these may be the most differentiating characteristics of sport participation for each sex [52,53,54]. The lack of differences in muscle mass and bone strength by youth sport participation patterns among females could also be partly explained by the high total body mass in the no-participation group: excess body mass supported by muscle mass may act as an extra chronic load (and hormonal profile) responsible for musculoskeletal adaptations of adolescents with obesity, especially in females [55,56]. We cannot, therefore, say that females are not responsive to anabolic stimuli such as sport participation, as shown in earlier IBDS analyses [57].

Our finding of a sex difference in the effects of youth sport participation on body fat is comparable to the results in the prior study [24], which reported that females who consistently participated in youth sports had lower waist circumferences in early adulthood as compared to females who did not participate or dropped out, while no difference was found by youth sport participation among males. However, caution is required in interpreting our results, as sex differences for body fat outcomes could be partly confounded by energy intake during childhood and adolescence. If males who consistently participated in youth sports concurrently had higher energy intakes than males who did not, this would have biased the association toward the null.

Our findings for sex-specific benefits of youth sport participation on body compartments have important implications. Greater bone and muscle mass without lower fat mass among males who consistently participated in youth sports would result in a higher BMI. Thus, the utilization of BMI for obesity evaluation could misclassify fit young adult males as being overweight or obese [58]. Achieving adequate muscle mass, which is different from having a low BMI, is important for optimal vascular health among young adult males [58]. Further, young males are not motivated to engage in an intervention for weight loss or weight gain prevention [59]. Taken together, in designing youth sports and physical activity programs for males, muscle mass or muscle strength, rather than weight or adiposity such as BMI, could be more appropriate targets. Conversely, among females, as consistent youth sport participation is associated with lower body fat in early adulthood, an intervention focusing on weight or adiposity outcomes would be appropriate.

It should be highlighted that the size of the effects of youth sport participation on body composition compartments was relatively large compared to the size of the effect of current MVPA. For example, the effect of consistent youth sport participation on BMC was equivalent to an additional 170 min/day of MVPA at age 23 years among males. The effect of consistent youth participation on fat mass was equivalent to an additional 30 min/day of MVPA at age 23 years among females. Given that individuals, on average, reduce physical activity in their transition to early adulthood while experiencing significant life events such as entering college and the labor market [60,61], it is unrealistic to anticipate a 30−170 min/day increase in MVPA among young adults. Therefore, the large effect of youth sport participation on body composition is unlikely to be compensated for by a MVPA increase in early adulthood. For females in particular, youth sport participation strongly predicts physical activity and other healthy habits in adulthood [36,62]. Therefore, promoting youth sport participation will be an effective strategy for establishing and maintaining healthy physical activity habits and achieving healthy body composition later in life [10,63,64,65,66,67].

The strengths of this study include the use of a three-body compartment model, a comprehensive assessment of longitudinal youth sport participation from age 6 to 17 years, and state-of-the-art outcome assessments using DXA and CT scans. However, some limitations should be acknowledged. The study did not account for the timing of sport participation drop-out, nor did it consider the effects of other forms of physical activity (e.g., active commuting to school) outside of organized sports during childhood and adolescence. Additionally, the analysis of subgroups with a small sample size may have been underpowered to detect medium-sized effects. Furthermore, unmeasured confounders, such as calcium or protein intake, psychological state, and stress or mental health during childhood and adolescence, may have introduced bias into the results.

Because loss to follow-up over this 23-year study may have caused selection bias, caution is required to generalize the results to the entire sample of the Iowa Fluoride Study or IBDS. The inclusion of mostly non-Hispanic White participants from a Midwestern US geographic region also limits the generalizability of the results to other population groups. The interscholastic sport participation measure did not consider weekly training sessions, the time of training sessions, the level of participation, or injuries throughout the sports career. Future research is warranted to understand potential sex-specific mechanisms by which youth sport participation influences body fat accumulation and identify sex-specific strategies to promote youth sport participation.

## 5. Conclusions

This study showed sex differences in the benefits of youth sport participation on early adulthood body composition, resulting in healthier bone and muscle for males and healthier body fat for females. Participation in high-power interscholastic sports, in particular, is beneficial for bone and muscle health in early adulthood. This study provides evidence to support the promotion of youth sport participation for healthy body composition later in life.

## Figures and Tables

**Table 1:** Comparison of study measurements by sex and youth sport participation trajectory groups.

	Male (*n* = 144)			Female (*n* = 184)		
Youth Sport Participation Group	Consistent Participation (*n* = 56)	Drop-Out (*n* = 53)	No Participation (*n* = 35)	Consistent Participation (*n* = 73)	Drop-Out (*n* = 66)	No Participation (*n* = 45)
	Mean ± SD	Mean ± SD	Mean ± SD	Mean ± SD	Mean ± SD	Mean ± SD
Birthweight, kg	3.6 ± 0.6	3.6 ± 0.6	3.5 ± 0.6	3.5 ± 0.5	3.4 ± 0.6	3.5 ± 0.5
Baseline BMC, g	285 ± 106	325 ± 136	328 ± 140	288 ± 108	302 ± 123	271 ± 105
Baseline BMI percentile	62 ± 27	62 ± 29	58 ± 30	59 ± 26	62 ± 28	60 ± 29
MVPA, min/d	27 ± 17	24 ± 16	28 ± 24	25 ± 17 *	23 ± 20 ^†^	14 ± 12 *^,†^
Height, cm	181 ± 8	181 ± 9	179 ± 7	167 ± 6 *	167 ± 8 ^†^	163 ± 7 *^,†^
Body mass, kg	94.2 ± 19.8	96.1 ± 22.6	87.2 ± 21.5	74.8 ± 18.4	77.0 ± 20.6	78.5 ± 24.6
BMI, kg/m^2^	28.6 ± 5.5	29.3 ± 6.5	27.0 ± 6.0	26.6 ± 5.9 *	27.5 ± 6.8	29.3 ± 8.7 *
BMC, g	2689 ± 481 *^,§^	2513 ± 425 ^†,§^	2280 ± 417 *^,†^	1908 ± 311 *	1812 ± 294	1735 ± 306 *
Lean mass, kg	66.3 ± 10.4 *	65.7 ± 11.3 ^†^	59.4 ± 10.6 *^,†^	45.1 ± 7.5	45.3 ± 8.3	44.2 ± 9.0
LMI, kg/m^2^	20.2 ± 2.5 *	20.1 ± 3.2 ^†^	18.4 ± 2.7 *^,†^	16.0 ± 2.2	16.2 ± 2.4	16.5 ± 3.1
Fat mass, kg	24.7 ± 11.4	27.4 ± 13.6	25.0 ± 12.0	27.3 ± 11.7	29.4 ± 13.0	32.1 ± 15.9
FMI, kg/m^2^	7.5 ± 3.4	8.3 ± 4.0	7.7 ± 3.6	9.7 ± 4.0 *	10.5 ± 4.6	12.0 ± 5.8 *
BMC-to-lean ratio, g/kg	40.6 ± 4.2 *^,§^	38.5 ± 4.1 ^§^	38.5 ± 3.8 *	42.5 ± 3.9 *^,§^	40.4 ± 4.6 ^§^	39.8 ± 5.0 *
Lean-to-fat ratio, kg/kg	3.04 ± 0.91 *	2.80 ± 0.95	2.72 ± 0.91 *	1.84 ± 0.54 *	1.76 ± 0.59	1.59 ± 0.51 *
Bone stiffness, kN/mm	715 ± 150 *	680 ± 139 ^†^	601 ± 112 *^,†^	515 ± 94	494 ± 97	499 ± 88

*p* < 0.05 * between consistent participation and no participation, ^†^ between drop-out and no participation, and ^§^ between consistent participation and drop-out; BMC, bone mineral content; BMI, body mass index; FMI, fat mass index; LMI, lean mass index; MVPA, moderate- and vigorous-intensity physical activity measured by ActiGraph accelerometers at age 23 years; SD, standard deviation.

**Table 2:** Bone and muscle measurements at age 23 years by sex and high school sport interscholastic participation groups.

	Male (*n* = 144)			Female (*n* = 184)		
	High-Power Sports (*n* = 62)	Other Sports (*n* = 20)	No Sports (*n* = 48)	High-Power Sports (*n* = 78)	Other Sports (*n* = 37)	No Sports (*n* = 61)
	Mean ± SD	Mean ± SD	Mean ± SD	Mean ± SD	Mean ± SD	Mean ± SD
BMC, g	2703 ± 454 *^,§^	2488 ± 491 ^§^	2325 ± 412 *	1919 ± 319 *^,§^	1768 ± 306 ^§^	1743 ± 281 *
LMI, kg/m^2^	20.1 ± 2.5	19.4 ± 2.9	19.3 ± 3.2	16.4 ± 2.3	15.8 ± 2.4	16.1 ± 2.8
BMC-to-lean ratio, g/kg	40.6 ± 4.0 *	39.5 ± 3.6	37.6 ± 4.0 *	41.6 ± 4.0	40.6 ± 4.5	40.4 ± 5.2
Lean-to-fat ratio, kg/kg	3.09 ± 0.91 *	2.98 ± 0.82	2.51 ± 0.96 *	1.76 ± 0.51	1.84 ± 0.66	1.71 ± 0.54
Bone stiffness, kN/mm	701 ± 138 *	692 ± 128	624 ± 140 *	513 ± 96	484 ± 80	496 ± 97

*p* < 0.05 * between high-power sports and no sports and ^§^ between high-power and other sports. No significant difference was found between other sports and no sports. BMC, bone mineral content; LMI, lean mass index; SD, standard deviation.

**Table 3:** Multivariable linear regression models for body composition compartments at age 23 years predicted by youth sport participation trajectory groups.

	Male						Female			
	BMC (g)		LBM (kg/m^2^)		Bone Stiffness (kN/mm)		BMC (g)		FMI (kg/m^2^) *	
Predictor	β ± SE	*p*	β ± SE	*p*	β ± SE	*p*	β ± SE	*p*	β ± SE	*p*
Intercept	−3849 ± 1183	<0.01	13.4 ± 7.1	0.06	145 ± 42	0.75	−1863 ± 759	0.01	9.5 ± 11.6	0.41
Baseline BMC (g)	1 ± 0.2	<0.01	NA	NA	0.01 ± 0.01	0.32	1 ± 0.1	<0.01	NA	NA
Baseline BMI percentile	NA	NA	0.01 ± 0.001	<0.01	NA	NA	NA	NA	0.1 ± 0.01	<0.01
Age (years)	−23 ± 44	0.60	0.05 ± 0.3	0.87	18 ± 20	0.37	−36 ± 28	0.20	−0.1 ± 0.5	0.76
Education: <4-year college vs. ≥4-year college	−47 ± 52	0.36	0.2 ± 0.4	0.64	−20 ± 23	0.40	−0.3 ± 33	0.99	1.4 ± 0.6	0.02
Height (cm)	35 ± 3	<0.01	NA	NA	NA	NA	26 ± 2	<0.01	NA	NA
Energy intake: highest vs. lowest tertile	NA	NA	NA	NA	NA	NA	NA	NA	−0.1 ± 0.6	0.87
Energy intake: middle vs. lowest tertile	NA	NA	NA	NA	NA	NA	NA	NA	1.1 ± 0.7	0.09
Additional 10 min/day of MVPA	21 ± 14	0.13	0.03 ± 0.1	0.72	8 ± 6	0.17	−2 ± 9	0.81	−0.5 ± 0.2	<0.01
Youth sports: consistent participation vs. no participation	377 ± 67	<0.01	1.6 ± 0.5	<0.01	112 ± 30	<0.01	58 ± 41	0.16	−1.4 ± 0.7	0.04
Youth sports: drop-out vs. no participation	192 ± 67	<0.01	1.5 ± 0.5	<0.01	76 ± 30	0.01	−46 ± 41	0.26	−1.3 ± 0.7	0.08

* Coefficients (β) and SE are presented after being back-transformed to the original unit from the regression model with the dependent variable of log-transformed FMI. BMC, bone mineral content; FMI, fat mass index; MVPA, moderate- and vigorous-intensity physical activity measured by ActiGraph accelerometers at age 23 years; NA, not applicable; SE, standard error.

**Table 4:** Multivariable linear regression models for ratios of body composition compartments at age 23 years predicted by youth sport participation trajectory groups.

	Male				Female			
	BMC-to-Lean Ratio (g/kg)		Lean-to-Fat Ratio (kg/kg)		BMC-to-Lean Ratio (g/kg)		Lean-to-Fat Ratio (kg/kg)	
Predictor	β ± SE	*p*	β ± SE	*p*	β ± SE	*p*	β ± SE	*p*
Intercept	40.4 ± 13.5	<0.01	3.42 ± 2.97	0.25	46.9 ± 13.3	<0.01	1.39 ± 1.45	0.34
Baseline BMI percentile	−0.03 ± 0.01	<0.01	−0.01 ± 0.003	<0.01	−0.06 ± 0.01	<0.01	−0.01 ± 0.001	<0.01
Age (years)	0.005 ± 0.6	0.99	−0.03 ± 0.13	0.84	−0.2 ± 0.6	0.79	0.03 ± 0.06	0.60
Education: <4-year college vs. ≥4-year college	−0.8 ± 0.7	0.21	−0.05 ± 0.15	0.75	−1.2 ± 0.7	0.07	−0.18 ± 0.07	0.01
Additional 10 min/day of MVPA	0.2 ± 0.2	0.28	0.16 ± 0.04	<0.01	0.1 ± 0.2	0.41	0.06 ± 0.02	<0.01
Youth sports: consistent participation vs. no participation	2.0 ± 0.9	0.02	0.36 ± 0.19	0.06	2.3 ± 0.8	<0.01	0.14 ± 0.09	0.11
Youth sports: drop-out vs. no participation	0.06 ± 0.9	0.95	0.18 ± 0.19	0.36	0.4 ± 0.8	0.60	0.12 ± 0.09	0.18

BMC, bone mineral content; BMI, body mass index; MVPA, moderate- and vigorous-intensity physical activity measured by ActiGraph accelerometers at age 23 years; SE, standard error.

## Data Availability

The data that support the findings of this study are openly available in dbGaP at https://www.ncbi.nlm.nih.gov/gap/ (accessed on 13 November 2023), reference number [phs002203].

## Supplementary Materials

The following supporting information can be downloaded at: https://www.mdpi.com/article/10.3390/ijerph22030416/s1, Table S1. Multivariable linear regression models for body compartments at age 23 years predicted by high school interscholastic sport participation groups.

## Abbreviations

The following abbreviations are used in this manuscript:

ANOVA	Analysis of variance
BMC	Bone mineral content
BMI	Body mass index
CT	Computed tomography
DXA	Dual-energy X-ray absorptiometry
FEA	Finite element analysis
FMI	Fat mass index
IBDS	Iowa Bone Development Study
LMI	Lean mass index
MVPA	Moderate- and vigorous-intensity physical activity
NYSS	National Youth Sports Strategy
PAQ	Physical Activity Questionnaire
SD	Standard deviation

## References

[1] Welk G., Janz K., Laurson K., Mahar M., Zhu W., Pavlovic A. (2022). Development of criterion-referenced standards for musculoskeletal fitness in youth: Considerations and approaches by the FitnessGram Scientific Advisory Board. Meas. Phys. Educ. Exerc. Sci..

[2] Xue S., Kemal O., Lu M., Lix L.M., Leslie W.D., Yang S. (2020). Age at attainment of peak bone mineral density and its associated factors: The National Health and Nutrition Examination Survey 2005–2014. Bone.

[3] Lindgren E., Rosengren B.E., Karlsson M.K. (2019). Does peak bone mass correlate with peak bone strength? Cross-sectional normative dual energy X-ray absorptiometry data in 1052 men aged 18–28 years. BMC Musculoskelet. Disord..

[4] Reid K.F., Fielding R.A. (2012). Skeletal muscle power: A critical determinant of physical functioning in older adults. Exerc. Sport Sci. Rev..

[5] Dulloo A.G., Bosy-Westphal A., Muller M.J. (2021). Diagnosis of obesity based on body composition-associated health risks—Time for a change in paradigm. Obes. Rev..

[6] Stephen W.C., Janssen I. (2009). Sarcopenic-obesity and cardiovascular disease risk in the elderly. J. Nutr. Health Aging.

[7] Zamboni M., Mazzali G., Fantin F., Rossi A., Di Francesco V. (2008). Sarcopenic obesity: A new category of obesity in the elderly. Nutr. Metab. Cardiovasc. Dis..

[8] Lee J.-H., Kim S.-Y., Kim D.-I. (2022). Association of muscle strength and body mass index with risk factors for metabolic syndrome and its prevalence in Korean adult women. BMC Public Health.

[9] Kim Y.H., So W.-Y. (2016). A low arm and leg muscle mass to total body weight ratio is associated with an increased prevalence of metabolic syndrome: The Korea National Health and Nutrition Examination Survey 2010–2011. Technol. Health Care.

[10] Kim S., Valdez R. (2015). Metabolic risk factors in U.S. youth with low relative muscle mass. Obes. Res. Clin. Pract..

[11] Peterson M.D., Zhang P., Saltarelli W.A., Visich P.S., Gordon P.M. (2016). Low Muscle Strength Thresholds for the Detection of Cardiometabolic Risk in Adolescents. Am. J. Prev. Med..

[12] Kim J.-H., Park Y.S. (2016). Low muscle mass is associated with metabolic syndrome in Korean adolescents: The Korea National Health and Nutrition Examination Survey 2009–2011. Nutr. Res..

[13] Zembura M., Matusik P. (2022). Sarcopenic Obesity in Children and Adolescents: A Systematic Review. Front. Endocrinol..

[14] Armas L.A.G., Lappe J.M., Heaney R.P. (2010). Calcium, Bone Strength and Fractures.

[15] Seeman E., Delmas P.D. (2006). Bone Quality—The Material and Structural Basis of Bone Strength and Fragility. N. Engl. J. Med..

[16] Friedman A.W. (2006). Important Determinants of Bone Strength: Beyond Bone Mineral Density. J. Clin. Rheumatol..

[17] Ott S.M. (2016). Bone strength: More than just bone density. Kidney Int..

[18] Nelson M.C., Story M., Larson N.I., Neumark-Sztainer D., Lytle L.A. (2008). Emerging adulthood and college-aged youth: An overlooked age for weight-related behavior change. Obesity.

[19] Gordon-Larsen P., The N.S., Adair L.S. (2010). Longitudinal trends in obesity in the United States from adolescence to the third decade of life. Obesity.

[20] Harris K.M., Perreira K.M., Lee D. (2009). Obesity in the transition to adulthood: Predictions across race/ethnicity, immigrant generation, and sex. Arch. Pediatr. Adolesc. Med..

[21] Gordon-Larsen P., Adair L.S., Nelson M.C., Popkin B.M. (2004). Five-year obesity incidence in the transition period between adolescence and adulthood: The National Longitudinal Study of Adolescent Health. Am. J. Clin. Nutr..

[22] Williamson D.F. (1990). The 10-year incidence of overweight and major weight gain in US adults. AMA Arch. Intern. Med..

[23] Katsoulis M., Lai A.G., Diaz-Ordaz K., Gomes M., Pasea L., Banerjee A., Denaxas S., Tsilidis K., Lagiou P., Misirli G. (2021). Identifying adults at high-risk for change in weight and BMI in England: A longitudinal, large-scale, population-based cohort study using electronic health records. Lancet Diabet Endocrinol..

[24] Logan K., Lloyd R.S., Schafer-Kalkhoff T., Khoury J.C., Ehrlich S., Dolan L.M., Schafer-Kalkhoff T., Khoury J.C., Ehrlich S., Dolan L.M. (2020). Youth sports participation and health status in early adulthood: A 12-year follow-up. Prev. Med. Rep..

[25] Zymbal V., Baptista F., Letuchy E.M., Janz K.F., Levy S.M. (2019). Mediating effect of muscle on the relationship of physical activity and bone. Med. Sci. Sports Exerc..

[26] Pashkova A., Hartman J.M., Letuchy E.M., Janz K.F. (2022). Interscholastic Athletics and Bone Strength: The Iowa Bone Development Study. J. Strength Cond. Res..

[27] Kwon S., Janz K., Letuchy E., Burns T., Levy S. (2015). Active lifestyle in childhood and adolescence prevents obesity development in young adulthood: Iowa Bone Development Study. Obesity.

[28] Guddal M.H., Stensland S.O., Smastuen M.C., Johnsen M.B., Heuch I., Zwart J.-A., Storheim K. (2020). Obesity in Young Adulthood: The role of physical activity level, musculoskeletal pain, and psychological distress inaAdolescence (The HUNT-Study). Int. J. Environ. Res. Public Health.

[29] McVeigh J.A., Howie E.K., Zhu K., Walsh J.P., Straker L. (2019). Organized Sport Participation From Childhood to Adolescence Is Associated With Bone Mass in Young Adults From the Raine Study. J. Bone Miner. Res..

[30] Ramires V.V., Dumith S.C., Wehrmeister F.C., Hallal P.C., Menezes A.M., Gonçalves H. (2016). Physical activity throughout adolescence and body composition at 18 years: 1993 Pelotas (Brazil) birth cohort study. Int. J. Behav. Nutr. Phys. Act..

[31] Hart N.H., Nimphius S., Rantalainen T., Ireland A., Siafarikas A., Newton R.U. (2017). Mechanical basis of bone strength: Influence of bone material, bone structure and muscle action. J. Musculoskelet. Neuronal Interact..

[32] Levy S.M., Kiritsy M.C., Slager S.L., Warren J.J. (1998). Patterns of dietary fluoride supplement use during infancy. J. Public Health Dent..

[33] Janz K.F., Burns T.L., Levy S.M., Iowa Bone Development Study (2005). Tracking of activity and sedentary behaviors in childhood: The Iowa Bone Development Study. Am. J. Prev. Med..

[34] Janz K.F., Kwon S., Letuchy E.M., Eichenberger Gilmore J.M., Burns T.L., Torner J.C., Levy S.M., Lytle L.A. (2009). Sustained effect of early physical activity on body fat mass in older children. Am. J. Prev. Med..

[35] U.S. Department of Health and Human Services (2019). National Youth Sports Strategy; Department of Health and Human Services.

[36] Kwon S., Letuchy E.M., Levy S.M., Janz K.F. (2021). Youth Sports Participation Is More Important among Females than Males for Predicting Physical Activity in Early Adulthood: Iowa Bone Development Study. Int. J. Environ. Res. Public Health.

[37] Kwon S., Janz K., Letuchy E., Trudy B., Steven L. (2015). Developmental trajectories of physical activity, sports, and television viewing during childhood to young adulthood. JAMA Pediatr..

[38] Kowlaski K., Crocker P., Faulkner R. (2007). Validation of the physical activity questionnaire for older children. Pediatr. Exerc. Sci..

[39] Kowlaski K., Crocker P., Kowlaski N. (1997). Convergent validity of the physical activity questionnaire for adolescents. Pediatr. Exerc. Sci..

[40] Nagin D., Odgers C. (2010). Group-based trajectory modeling in clinical research. Annu. Rev. Clin. Psychol..

[41] Nilsson M., Ohlsson C., Mellström D., Lorentzon M. (2013). Sport-specific association between exercise loading and the density, geometry, and microstructure of weight-bearing bone in young adult men. Osteoporos. Int..

[42] Nikander R., Sievänen H., Heinonen A., Daly R.M., Uusi-Rasi K., Kannus P. (2010). Targeted exercise against osteoporosis: A systematic review and meta-analysis for optimising bone strength throughout life. BMC Med..

[43] Ward R.C., Janz K.F., Letuchy E.M., Peterson C., Levy S.M. (2018). Contribution of High School Sport Participation to Young Adult Bone Strength. Med. Sci. Sports Exerc..

[44] Messina C., Albano D., Gitto S., Tofanelli L., Bazzocchi A., Ulivieri F.M., Gitto S., Tofanelli L., Bazzocchi A., Ulivieri F.M. (2020). Body composition with dual energy X-ray absorptiometry: From basics to new tools. Quant. Imaging Med. Surg..

[45] Baptista F., Zymbal V., Janz K.F. (2022). Predictive Validity of Handgrip Strength, Vertical Jump Power, and Plank Time in the Identification of Pediatric Sarcopenia. Meas. Phys. Educ. Exerc. Sci..

[46] Bevill G., Keaveny T.M. (2009). Trabecular bone strength predictions using finite element analysis of micro-scale images at limited spatial resolution. Bone.

[47] MacNeil J.A., Boyd S.K. (2007). Accuracy of high-resolution peripheral quantitative computed tomography for measurement of bone quality. Med. Eng. Phys..

[48] Engelke K., van Rietbergen B., Zysset P. (2016). FEA to Measure Bone Strength: A Review. Clin. Rev. Bone Miner. Metab..

[49] Troiano R.P., Berrigan D., Dodd K.W., Masse L.C., Tilert T., McDowell M. (2008). Physical activity in the United States measured by accelerometer. Med. Sci. Sports Exerc..

[50] Ubago-Guisado E., Mata E., Sanchez-Sanchez J., Plaza-Carmona M., Martin-Garcia M., Gallardo L. (2017). Influence of different sports on fat mass and lean mass in growing girls. J. Sport Health Sci..

[51] Bielemann R.M., Martinez-Mesa J., Gigante D.P. (2013). Physical activity during life course and bone mass: A systematic review of methods and findings from cohort studies with young adults. BMC Musculoskelet. Disord..

[52] Tipton K.D. (2001). Gender differences in protein metabolism. Curr. Opin. Clin. Nutr. Metab. Care.

[53] Jansson D., Lindberg A.-S., Lundberg E., Domellöf M., Theos A. (2022). Effects of Resistance and Endurance Training Alone or Combined on Hormonal Adaptations and Cytokines in Healthy Children and Adolescents: A Systematic Review and Meta-analysis. Sports Med. Open.

[54] Baxter-Jones A.D.G., Jackowski S.A. (2021). Sex differences in bone mineral content and bone geometry accrual: A review of the Paediatric Bone Mineral Accural Study (1991–2017). Ann. Hum. Biol..

[55] Winther A., Jørgensen L., Ahmed L.A., Christoffersen T., Furberg A.-S., Grimnes G., Jørgensen L., Ahmed L.A., Christoffersen T., Furberg A.-S. (2018). Bone mineral density at the hip and its relation to fat mass and lean mass in adolescents: The Tromsø Study, Fit Futures. BMC Musculoskelet. Disord..

[56] Garcia-Vicencio S., Martin V., Chalchat E., Penailillo L., Kluka V., Fourot A.V., Penailillo L., Kluka V., Fourot A.V., Moreira M.B. (2023). Sex-Related Neuromuscular Adaptations to Youth Obesity: Force, Muscle Mass, and Neural Issues. Adv. Exp. Med. Biol..

[57] Metcalf K.M., Letuchy E.M., Levy S.M., Janz K.F. (2020). An 8-Year Longitudinal Analysis of Physical Activity and Bone Strength From Adolescence to Emerging Adulthood: The Iowa Bone Development Study. Pediatr. Exerc. Sci..

[58] Agbaje A.O., Barker A.R., Tuomainen T.-P. (2023). Cumulative muscle mass and blood pressure but not fat mass drives arterial stiffness and carotid intima-media thickness progression in the young population and is unrelated to vascular organ damage. Hypertens. Res..

[59] Lanoye A., Brown K.L., LaRose J.G. (2017). The Transition into Young Adulthood: A Critical Period for Weight Control. Curr. Diab. Rep..

[60] Gropper H., John J.M., Sudeck G., Thiel A. (2020). The impact of life events and transitions on physical activity: A scoping review. PLoS ONE.

[61] Corder K., Winpenny E., Love R., Brown H.E., White M., Sluijs E.V. (2019). Change in physical activity from adolescence to early adulthood: A systematic review and meta-analysis of longitudinal cohort studies. Br. J. Sports Med..

[62] Palomäki S., Hirvensalo M., Smith K., Raitakari O., Männistö S., Hutri-Kähönen N., Tammelin T. (2018). Does organized sport participation during youth predict healthy habits in adulthood? A 28-year longitudinal study. Scand. J. Med. Sci. Sports.

[63] Howie E.K., Daniels B.T., Guagliano J.M. (2018). Promoting Physical Activity Through Youth Sports Programs: It’s Social. Am. J. Lifestyle Med..

[64] Allender S., Cowburn G., Foster C. (2006). Understanding participation in sport and physical activity among children and adults: A review of qualitative studies. Health Educ. Res..

[65] Von Seggern M.J., Rogers A.E., Schenkelberg M.A., Kellstedt D.K., Welk G.J., High R., Dzewaltowski D.A. (2024). Sociodemographic influences on youth sport participation and physical activity among children living within concentrated Hispanic/Latino rural communities. Front. Public Health.

[66] Flaherty M., Sagas M. (2021). Shifting the Paradigm: A Constructivist Analysis of Agency and Structure in Sustained Youth Sport Participation. Front. Psychol..

[67] Grady C.L., Murtagh E., Ng K., Bengoechea E.G., Woods C.B. (2025). Communicating physical activity messages with adolescents: What works? A scoping review with stakeholder consultation. Int. J. Behav. Nutr. Phys. Act..

