# Supplementary material for: Associations Between Youth Sport Participation and Bone, Muscle, and Fat in Adulthood: Iowa Bone Development Study
Source: Int J Environ Res Public Health. 2025 Mar 12;22(3):416. doi: 10.3390/ijerph22030416 (PMC11942570; doi:10.3390/ijerph22030416)
Supplement: Supplementary file 1 [file ijerph-22-00416-s001.zip › ijerph-3475609-supplementary.pdf]

Supplementary Data

Supplementary Table 1. Multivariable linear regression models for body compartments at age 23 years predicted by high school interscholastic sport participation groups.

	Male								Female	
	BMC (g)		BMC-to-lean ratio (g/kg)		Lean-to-fat ratio (kg/kg)		Bone stiffness (kN/mm)		BMC (g)	
Predictor	β±SE	*p*	β±SE	*p*	β±SE	*p*	β±SE	*p*	β±SE	*p*
Intercept	-4360±1265	<0.01	38.5±13.8	<0.01	4.23±3.04	0.16	-2.8±489	0.99	-2245±767	0.41
Baseline BMC (g)	1±0.2	<0.01	NA	NA	0.01±0.003	<0.01	-0.01±0.1	0.94	1±0.2	<0.01
Baseline BMI percentile	NA	NA	-0.03±0.01	<0.01	NA	NA	NA	NA	NA	NA
Age (years)	6±48	0.89	0.03±0.6	0.95	-0.07±0.13	0.61	27±21	0.20	-21±28	0.76
Education:<4-year college vs. ≥4-year college	-93±54	0.09	-1.0±0.7	0.14	-0.006±0.15	0.97	-34±24	0.16	0.3±34	0.02
Height (cm)	35±3	<0.01	NA	NA	NA	NA	NA	NA	26±2	
Additional 10 minutes/day of MVPA	23±14	0.13	0.3±0.2	0.14	0.15±0.04	<0.01	8±6	0.18	-2±9	<0.01
Interscholastic sports: high-power sports vs. no sports	254±60	<0.01	3.1±0.7	<0.01	0.62±0.16	<0.01	73±26	<0.01	70±37	0.06
Interscholastic sports: other sports vs. no sports	123±81	0.13	1.5±1.0	0.14	0.40±0.22	0.07	58±36	0.11	-14±43	0.74

BMC, whole body bone mineral content; FMI, fat mass index; LBMI, lean body mass index; MVPA, moderate- and vigorous-intensity physical activity measured by ActiGraph accelerometers at age 23 years; NA, not applicable; SE, standard error.
